# Editorial: Pharmacogenomics for improving drug safety and efficacy in cancer

**DOI:** 10.3389/fphar.2025.1649258

**Published:** 2025-07-01

**Authors:** Zhiyao He, Xuewei Fu, Zhijie Xu, Omar Alshargi, Roberto Rodríguez-Labrada, Xiao-Bo Zhong

**Affiliations:** ^1^ Department of Pharmacy, West China Hospital, Sichuan University, Chengdu, China; ^2^ Key Laboratory of Drug-Targeting and Drug Delivery System of the Education Ministry and Sichuan Province, Sichuan Research Center for Drug Precision Industrial Technology, West China School of Pharmacy, Sichuan University, Chengdu, China; ^3^ Mental Health Center, West China Hospital, Sichuan University, Chengdu, China; ^4^ Department of Pathology, Xiangya Hospital, Central South University, Changsha, China; ^5^ College of Pharmacy, Riyadh Elm University, Riyadh, Saudi Arabia; ^6^ Department of Clinical Neurophysiology, Centre for the Research and Rehabilitation of Hereditary Ataxias, Holguín, Cuba; ^7^ Department of Pharmaceutical Sciences, School of Pharmacy, University of Connecticut, Storrs, United States

**Keywords:** pharmacogenomics, drug safety, precision oncology, cancer pharmacotherapy, genetic variability

The advancement of precision oncology has gradually reshaped cancer treatment paradigms, with pharmacogenomics emerging as a potentially valuable component of therapeutic approach (Hu et al., 2024; Jin and Zhong, 2023; Mondello et al., 2024). By identifying genetic variations that may influence drug response and toxicity, pharmacogenomic research aims to support more tailored therapeutic strategies, potentially enhancing efficacy while reducing adverse effects (Li et al., 2024; Qahwaji et al., 2024; Shriver et al., 2024). This Research Topic was developed to examine current progress in translating pharmacogenomic knowledge into clinical applications that could optimize drug selection, mitigate toxicity, and address resistance in cancer therapy.

Understanding underlying mechanisms remains essential for many pharmacogenomic insights (Chen et al., 2017). Casotti et al. explored methotrexate (MTX) resistance in high-grade osteosarcoma through a multimodal targeted next-generation sequencing approach. Their identification of single nucleotide polymorphisms (SNPs) in the folate pathway and transporter genes, alongside a novel DHFR-MSH3 fusion transcript, provided initial evidence on genomic and transcriptomic factors potentially contributing to MTX resistance. These findings provide candidate biomarkers for prediction of drug resistance and possible therapeutic targets, underscoring the need for further functional studies to clarify the impact of genetic alterations beyond the DNA sequence.

In pediatric oncology, individualized dosing remains critical due to developmental variability in drug metabolism (Vander Schaaf, et al., 2024). Choi et al. performed whole-exome sequencing in a Korean child population with acute lymphoblastic leukemia to identify genetic variants associated with delayed clearance of high-dose MTX, a known risk factor for nephrotoxicity. Some candidate SNPs in *ENG* and *PKD1L2* genes were identified, suggesting possible germline influences on drug elimination. This study points to the potential utility of exome-wide pharmacogenomic screening in pediatric populations but also calls for larger studies to validate these associations and integrate them into dosing guidelines.

Liu et al. demonstrated how pharmacogenomic approaches could be incorporated into clinical surveillance by adapting a trigger tool-based on the Global Trigger Tool to enhance detection of chemotherapy-related adverse drug events (ADEs) among hospitalized Chinese patients. In their retrospective analysis of 500 patients, the tool exhibited high sensitivity (91.8%) and identified ADEs in 63.0% of patients, primarily affecting hematologic and gastrointestinal systems. Multivariate analysis suggested that polypharmacy and previous ADEs significantly increased the risk for overall ADEs and serious adverse events. These results highlight the potential value of integrating pharmacogenomic screening with real-time monitoring to improve drug safety management in oncology, though prospective validation is warranted.

Zheng et al. systematically reviewed 83 studies investigating genetic polymorphisms associated with hematologic toxicity from platinum-based chemotherapy. Despite heterogeneity and some methodological limitations, 11 SNPs across 10 genes, including variants in *GSTP1*, *ERCC1*, and *XRCC1*, were consistently implicated in multiple populations. These findings identify promising genetic markers for predicting toxicity risk yet emphasize the need for further validation before routine clinical implementation.

The practical relevance of pharmacogenomics in real-world settings is illustrated by case reports included in this Research Topic. Zhang et al. involved a 48-year-old female with lung adenocarcinoma harboring coexisting *BRAF* V600E and *BRCA2* germline mutations, detected via dynamic molecular profiling including liquid biopsy after multiple treatment failures. Treatment with dabrafenib plus trametinib led to a rapid partial response, highlighting the potential of ongoing molecular monitoring to inform personalized therapies. However, the limited efficacy of PARP inhibitors in this *BRCA2*-mutant non-small cell lung cancer (NSCLC) case indicates a need for further study on their role outside classic BRCA-driven cancers.

Another case reported by Wang et al. described a patient with *HER2*-mutant NSCLC who showed a durable response to trastuzumab deruxtecan (DS-8201) following failure of first-line immunochemotherapy. The patient also developed delayed immune-related adrenal insufficiency, raising questions about the drug’s immunomodulatory effects. This case supports further investigation into the efficacy and safety of DS-8201, particularly in the context of prior immune checkpoint blockade.

Liang et al. provided a comprehensive review of antibody-drug conjugates (ADCs) in breast cancer, focusing on agents such as trastuzumab emtansine (T-DM1), trastuzumab deruxtecan (T-DXd), and sacituzumab govitecan (SG). This review discussed key elements influencing ADC function, including target antigen specificity, cytotoxic payloads, and linker strategies-alongside clinical trial outcomes and safety profiles. By discussing how genomic markers might guide patient selection and toxicity monitoring, the authors suggest that pharmacogenomics could contribute to refining ADC application across molecular subtypes, though further research is needed to confirm clinical utility.

In addition, Wang et al. retrospectively evaluated the efficacy and safety of the 5-fluorouracil/cisplatin/vincristine (FPV) chemotherapy regimen in patients with high-risk gestational trophoblastic neoplasia (GTN). Emphasizing the need to balance efficacy and toxicity in individualized treatment, the study found that the FPV regimen demonstrated favorable outcomes with manageable toxicity, showing comparable response and survival rates to the 5-fluorouracil/actinomycin D/vincristine (FAV) regimen. These findings suggest that future incorporation of pharmacogenomic markers might assist in optimizing regimen selection for GTN, pending further research.

In summary, the articles in this Research Topic address various aspects of pharmacogenomics in oncology, ranging from the identification of genetic variants influencing drug resistance and toxicity, the evaluation of novel targeted therapies and antibody-drug conjugates, to the clinical assessment of chemotherapy regimens. Together, they underscore both the potential benefits and current limitations of applying pharmacogenomic insights in cancer treatment, highlighting the need for further functional validation, prospective studies, and careful integration into clinical practice.
